# Genomic Analysis of Molecular Bacterial Mechanisms of Resistance to Phage Infection

**DOI:** 10.3389/fmicb.2021.784949

**Published:** 2022-02-17

**Authors:** Antón Ambroa, Lucia Blasco, María López, Olga Pacios, Inés Bleriot, Laura Fernández-García, Manuel González de Aledo, Concha Ortiz-Cartagena, Andrew Millard, María Tomás

**Affiliations:** ^1^Microbiology Department-Research Institute Biomedical A Coruña (INIBIC), Hospital A Coruña (CHUAC), University of A Coruña (UDC), A Coruña, Spain; ^2^Study Group on Mechanisms of Action and Resistance to Antimicrobials (GEMARA) the Behalf of the Spanish Society of Infectious Diseases and Clinical Microbiology (SEIMC), Madrid, Spain; ^3^Spanish Network for Research in Infectious Diseases (REIPI), Infectious Diseases Network Biomedical Research Center (CIBERINFEC), Carlos III Health Institute, Madrid, Spain; ^4^Department of Genetics and Genome Biology, University of Leicester, Leicester, United Kingdom

**Keywords:** bacterial, resistance, genomic island, phages, *Acinetobacter baumannii*, CRISPR, WGS or whole-genome sequencing

## Abstract

To optimize phage therapy, we need to understand how bacteria evolve against phage attacks. One of the main problems of phage therapy is the appearance of bacterial resistance variants. The use of genomics to track antimicrobial resistance is increasingly developed and used in clinical laboratories. For that reason, it is important to consider, in an emerging future with phage therapy, to detect and avoid phage-resistant strains that can be overcome by the analysis of metadata provided by whole-genome sequencing. Here, we identified genes associated with phage resistance in 18 *Acinetobacter baumannii* clinical strains belonging to the ST-2 clonal complex during a decade (Ab2000 vs. 2010): 9 from 2000 to 9 from 2010. The presence of genes putatively associated with phage resistance was detected. Genes detected were associated with an abortive infection system, restriction–modification system, genes predicted to be associated with defense systems but with unknown function, and CRISPR-Cas system. Between 118 and 171 genes were found in the 18 clinical strains. On average, 26% of these genes were detected inside genomic islands in the 2000 strains and 32% in the 2010 strains. Furthermore, 38 potential CRISPR arrays in 17 of 18 of the strains were found, as well as 705 proteins associated with CRISPR-Cas systems. A moderately higher presence of these genes in the strains of 2010 in comparison with those of 2000 was found, especially those related to the restriction–modification system and CRISPR-Cas system. The presence of these genes in genomic islands at a higher rate in the strains of 2010 compared with those of 2000 was also detected. Whole-genome sequencing and bioinformatics could be powerful tools to avoid drawbacks when a personalized therapy is applied. In this study, it allows us to take care of the phage resistance in *A. baumannii* clinical strains to prevent a failure in possible phage therapy.

## Introduction

As part of the ESKAPE pathogens, *Acinetobacter baumannii* is frequently isolated from infections in clinical environments, and its resistance against multiple antibiotics is increasingly common (Boral et al., 2019). For this reason, it is necessary to opt for alternative treatments, such as phage therapy. However, the ability of bacteria to develop resistance mechanisms against phages is possible, even when there is no previous treatment with phage therapy due to the constant coevolutionary interactions (Stern and Sorek, 2011). The spread of phage resistance presents a significant challenge to the efficacy of the therapy (Schooley et al., 2017; Oechslin, 2018).

It is important to know and characterize the phage resistance mechanisms of a certain species, clone, or strain, before phage treatment to minimize treatment failure. Whole-genome sequencing (WGS) has been demonstrated to be a powerful tool in the detection of phage resistance mechanisms, as well as the evolution of CRISPR-Cas arrays in bacteria subjected to phage pressure (Bishop-Lilly et al., 2012; Broniewski et al., 2020). WGS is increasingly becoming a cheaper and faster technology; thus, it is implemented progressively in routine hospital diagnostics and research (Mintzer et al., 2019).

Recently, new or modified phage resistance mechanisms have been discovered and characterized (Labrie et al., 2010). Although a large part of defense systems against phages are maintained over generations, there is a continuous emergence of resistance mechanisms due to spontaneous mutations as a consequence of the coexistence of phage and bacteria. Most of these mutations occur in the phage receptor proteins, used by the phages to adhere to the cell (Labrie et al., 2010). Moreover, recent publications have demonstrated that variations in genes often associated with mediating biosynthesis on the surface of bacteria are also related to phage resistance in *A. baumannii*, such as glucosyltransferases (Gordillo Altamirano et al., 2021; Wang et al., 2021). In recent years, phage resistance mechanisms have been attracting an increasing interest due to the rising knowledge in phage interactions with bacteria. This leads to the discovery and characterization of new phage resistance mechanisms such as Zorya, Druantia, or Thoeris (Doron et al., 2018). Phage resistance mechanisms are typically clustered in genomic “defense islands.” mobile genetic evolutionary elements that contain genes associated with phage defense systems (Krupovic et al., 2014; Doron et al., 2018).

**FIGURE 1:**
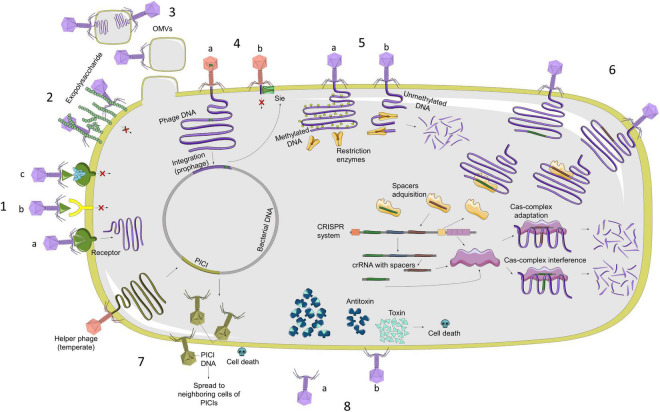
Representation of main mechanisms of bacterial resistance against phage infection (from left in a clockwise sense). 1. **(A)** Phage recognizes bacterial membrane receptor and can carry out infection; **(B)** alterations in receptors are produced by mutations and prevent phage from recognizing receptor, so it Will not infect bacteria; **(C)** bacteria can block recognition by producing inhibitors that bind to receptors. 2. Production of exopolysaccharide or extracellular matrix. 3. OMVs are composed of membrane lipids, membrane proteins, and periplasmic components. Some bacterial species use them as a decoy against phages as a defense mechanism. 4. **(A)** Bacteria block injection of DNA from other phages, acquiring Sie systems through prophages with this type of protein; **(B)** once bacterium has prophage in its genome with proteins that code for Sie system, it will be able to block entry of DNA from other phages. 5. **(A)** R–M system distinguishes between methylated and unmethylated DNA. Restriction enzymes cannot cut methylated DNA, which is also a protection system for bacterial DNA; **(B)** if phage DNA is not methylated, this system can cut injected DNA. 6. CRISPR-Cas system is an adaptative defense system, which recognizes phage DNA sequences, incorporates them into system, and then produces enzymes that are capable of recognizing these sequences to cut them. 7. PICI system is found in bacterial genome and induced by helper prophages to produce mature phage particles that assemble PICI system itself to kill infected cells and to be able to spread this system to adjacent cells. 8. **(A)** One of most characterized Abi systems is toxin–antitoxin system. Under normal conditions, bacterium expresses both proteins equally, so cell death does not occur; **(B)** When organism is subjected to stress situations, such as phage infection, toxin is highly expressed in comparison with antitoxin, causing cell death, which consequently produces a reduction in phage replication.

The main resistance mechanisms are related to the inhibition of the phage adsorption, blocking of phage DNA injection, cutting of the injected DNA, inhibition of the phage DNA replication, interference in the phage assembly, and bacterial suicide (Azam and Tanji, 2019). In Figure 1, we summarized all the characterized phage resistance mechanisms. In this study, we focused on those which could be bioinformatically detected without any experimental process:

i) Abortive Infection (ABI) systems, characterized by the fact that the phage enters the cell, but its development is interrupted in any phase (replication, transcription, or translation). The mechanism of action is not entirely clear, either because of their complexity or because they are widely varied from one species to another (Lopatina et al., 2020).
ii) Toxin/antitoxin (TA) systems are a specific type of ABI system, but they are well-characterized and widespread through diverse species (Harms et al., 2018). In this system, a toxin is produced by the cell and is neutralized by an antitoxin. The expression of these molecules is highly controlled and varies from one system to another. When the balance between one molecule and the other is disturbed, the toxin is released, and the bacteria die (Song and Wood, 2020).
iii) Restriction–Modification (R–M) systems consist of a restriction endonuclease and a methyltransferase. This type of system distinguishes the DNA of the host from foreign DNA to recognize and destroy phage DNA after its injection into the cell. When unmethylated phage DNA enters a bacterium that possesses the R–M system, it will be cleaved by the restriction endonuclease or methylated by the methyltransferase to escape the restriction (Vasu and Nagaraja, 2013).
iv) CRISPR-Cas (clustered regularly interspaced short palindromic repeats—CRISPR-associated proteins) system is an adaptative immune system that bacteria develop against phage DNA/RNA and other foreign DNA (Mojica and Rodriguez-Valera, 2016). The typical structure of the CRISPR-Cas locus is a leader sequence, followed by the repeat-spacer array and the *cas* genes operon (Guan et al., 2016). The adaptation of the CRISPR-Cas system is due to the acquisition of the spacer sequences, which are small fragments of foreign nucleic acids, between the repeats of the CRISPR locus (Guan et al., 2016). The functioning of the CRISPR-Cas system, usually divided into three steps (adaptation, processing, and guidance of the crRNA-CRISPR RNA- and targeting and interference of the foreign DNA/RNA), is carried out by the Cas (CRISPR-associated) proteins (Barrangou, 2013). CRISPR-Cas systems are classified according to their conserved *cas* genes and the architecture of the *cas* operon (Makarova et al., 2011). Until recently, little data existed about CRISPR-Cas systems in *A. baumannii*. The pangenome analysis of *A. baumannii* has shown CRISPR-Cas systems in the species (Mangas et al., 2019). One of the most characterized systems in *A. baumannii* is the CRISPR I-Fb system (Karah et al., 2015). However, most of the Cas-related genes and CRISPR arrays are yet not identified and characterized.

In this study, we searched for putative genes associated with phage resistance, and we focused on CRISPR-Cas systems by studying the CRISPR arrays and Cas protein presence through a bioinformatic approach in 18 genomes of clinical strains of *A. baumannii* isolated in the “II Spanish Study of *A. baumannii* GEIH-REIPI 2000–2010.”

## Methodology

### Genome Database

Eighteen clinical *A. baumannii* genomes previously sequenced and annotated (II Spanish Multicenter Study, GEIH-REIPI *Acinetobacter baumannii* 2000–2010; Umbrella Bioproject PRJNA422585) (López et al., 2016, 2017) have been studied. Nine strains were from 2000 and nine from 2010. All of the strains belong to the ST-2 clone (López et al., 2016).

### Search for General Genes Associated With Bacteriophage Resistance and Their Presence in Genomic Islands

To analyze the presence of genes putatively associated with phage resistance systems, a custom database based on genes from the ‘‘PADS Arsenal database’’^1^ was created (Zhang et al., 2020). The genes were grouped in five systems: ABI systems related (not belonging to toxin/antitoxin system), TA systems related, R–M system related, CRISPR-Cas-associated proteins, and newly (NEW) characterized systems-related genes. In this last category, we included those genes which hit against known phage-resistant genes but were associated with genes predicted to be associated with phage-resistant functions and whose function in *A. baumannii* is not clear yet, such as newly characterized systems (e.g., Zorya, Druantia, and Thoeris). In this category, it is also included those genes related to known and characterized systems but without a complete functional characterization in *A. baumannii* (e. g. BREX). A blast search of the complete genomes against this database and filtered out those hits with an *e*-value > 1E-04 was made. Statistical significance was determined by comparing the absolute number of each system in the nine genomes of Ab_GEIH-2000 collection against the absolute numbers of genes of Ab_GEIH-2010 collection with the Student’s *t*-test. The percentage of the genes involved in resistance was calculated by dividing the genes predicted to be associated with phage resistance by the total number of genes in the bacteria genome.

To locate genomic islands (GIs), three different approaches were used: IslandViewer with default settings (Bertelli et al., 2017), blast search with default settings and cutoff of *e*-value < 1e-03 against a previously constructed ICEberg database (Liu et al., 2019), and checking the GC content of the contigs of each genome (Zhang R. et al., 2014). The previously detected phage-resistant genes were localized in the GIs detected per genome, and an average percentage of genes by collection was calculated. Statistical significance was determined by comparing the absolute number of each system present in GIs in the nine genomes of Ab_GEIH-2000 collection against the absolute numbers of genes of Ab_GEIH-2010 collection with Student’s *t*-test.

### Search and Characterization of Clustered Regularly Interspaced Short Palindromic Repeats Arrays

On a first try, CRISPRCasFinder (Couvin et al., 2018) was used, but no putative CRISPR-Cas system was found. For this reason, CRISPR arrays were found using the CRISPR Recognition Tool (CRT) (Bland et al., 2007). The modification proposed by Rho et al. (2012) for whole-metagenomic assembled genomes called metaCRT was used with the following parameters: minimum number of repeats: 3, minimum repeat length: 12, maximum repeat length: 70, minimum spacer length: 18, maximum spacer length: 80, and with a search window: 6. This search pretends to be as flexible as possible because genomes are in contig format. Most existing CRISPR identification tools use 11 as the minimum word size for the repeat, as it does not compromise the finding of potential short repeats with multiple base mismatches (Biswas et al., 2016). Regarding spacer length, a new type of V-C CRISPR-Cas has a preference for short (17–19 bp) DNA fragments to incorporate as spacers (Wright et al., 2019); thereby, a minimum spacer length of 18 nt was covenanted.

**FIGURE 2:**
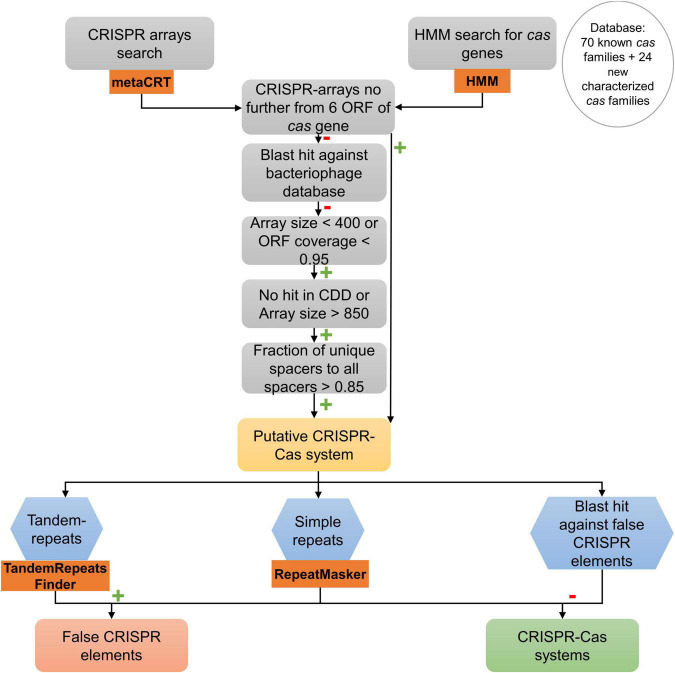
Search procedure of CRISPR-Cas systems in 18 genomes of *A. baumannii* clinical strains. Orange rectangle represents bioinformatics programs used for that task. Green plus marks represent a positive result for each operation. Red minus marks represent a negative result for each operation.

To filter and validate the CRISPR arrays, a similar procedure to the first step in the protocol developed by Shmakov et al. (2020) was followed (Figure 2). In the first step, CRISPR arrays separated by no more than six open reading frames (ORFs) to a putative Cas protein identified before were considered to be part of a putative CRISPR-Cas system. Those which were not part of a putative CRISPR-Cas system were considered to be single possible CRISPR arrays. To validate these arrays, we made a short-blastn search of the spacers of the possible CRISPR arrays against all phage genomes using the INPHARED database (Cook et al., 2021). Results that were > 95% in identity and those arrays whose query hit was larger than 20 were considered putative CRISPR arrays.

Negative blast hits of the spacers of the CRISPR arrays against bacteriophage follow the procedure described by Shmakov et al. (2020) for isolated arrays: first, arrays > 400 bp and with an ORF coverage > 0.95 were filtered out. Second, all arrays with < 850 bp that had the domain in CDD search were filtered out (Marchler-Bauer et al., 2015; Lu et al., 2020). Then, pairwise distances between spacers of each array were calculated (number of matches in the longest blastn hit between them, divided by the length of the smaller spacer in the pair). The spacers of each array were clustered using single linkage clustering following the same procedure as Shmakov et al. (2020) with a cutoff of 0.3. A spacer similarity index was calculated for each CRISPR array as the number of clusters formed divided by the number of spacers in the array (1 means that all the spacers are different). Those arrays whose spacer similarity index was < 0.85 were filtered out. The rest were considered putative CRISPR arrays.

To complement the procedure made by Shmakov et al. (2020), a search about common special low-complexity sequences that may be confused as CRISPR arrays was made, known as false-CRISPR elements (Zhang and Ye, 2017). The presence of tandem repeats, potentially hypermutable regions that enable bacteria to adapt to evolving environments without increasing their mutation rate, was checked with Tandem Repeat Finder (Benson, 1999; Rando and Verstrepen, 2007; Zhou et al., 2014). The presence of short low-complexity repeats was also examined with RepeatMasker (Chen, 2004). To test the results and to complete the search of low-complexity sequences, a blast search against an existing false-CRISPR elements database was obtained from the CRISPRone website (Zhang and Ye, 2017).

The search for possible Cas-related proteins was made, based on the method of Zhang Q. et al. (2014), but adding a search in the “HMMCAS” website of all of their available HMM models, performed with a cutoff of reported *e*-values of Chai et al. (2019). The 18 genomes were examined using hmmscan with all of the pfam HMM profiles based on National Center for Biotechnology Information entries of known Cas protein families searching in the pfam database 70 Cas-related protein families and other CRISPR-associated proteins in the pfam database (e.g., DEAD/DEAH box helicase), 93 families that were in the TIGRFAMS resource, and the 24 newly characterized families (Haft et al., 2003; Finn et al., 2011, 2014; Zhang Q. et al., 2014). For the TIGRFAMS database proteins, it was necessary to build an HMM profile with hmmbuild with default settings (Finn et al., 2011), making a previous alignment for each compound of proteins with Clustal Omega version 1.2.4 (ClustalO) (Sievers and Higgins, 2014).

All of the input and output data for the searches (genes associated with phage resistance, genomic islands, CRISPR arrays, and Cas proteins) were processed with Python^2^ and BioPython (19304878; Chapman and Chang, 2000; Cock et al., 2009).

To establish the evolution and to compare the presence of the CRISPR arrays among the same clonal complex of the 18 clinical strains of *A. baumannii*, a phylogenetic tree was made using the CRISPR spacers detected. Trees were built using MEGA7 with CLUSTALW alignment (MEGA version 7.9.26) (Kumar et al., 1994, 2016; Thompson et al., 1994).

## Results

### Genes Putatively Associated With Phage Resistance in *Acinetobacter baumannii* Clinical Strains and Their Presence in Genomic Islands

**FIGURE 3:**
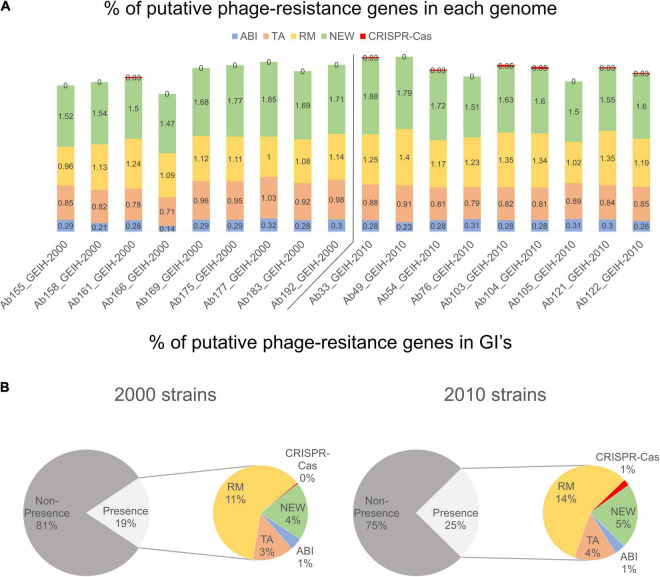
**(A)** Frequency (%, rounded to two decimal numbers) of each group of genes in each genome with respect to total of genes: abortive infection (ABI) system, toxin/antitoxin (TA) system, restriction–modification (R–M) system, and genes associated with newly phage resistance bacterial mechanisms (NEW), e.g., Zorya, Hachiman, and Druantia. **(B)** Presence and non-presence of putative phage-resistant genes in GIs. Presence (%, rounded without decimal numbers) section is divided into different groups of genes.

Between 118 and 171 genes were detected per genome; those could be putatively associated with bacterial defense against bacteriophages (Supplementary Table 1). The frequency (%) of each resistance system was calculated with the number of genes of each group divided by the total genes per genome. It was observed that the genes related to R–M and CRISPR-Cas systems showed a slightly significantly higher prevalence in 2010 strains (Figure 3A) (*p* < 0.024 and *p* < 0.0144, respectively). The frequency of the genes related to ABI, TA, and new systems remained constant in both collections. The presence of putative phage-resistant genes in GIs was also predicted (Supplementary Table 2), and it was found that GIs represent in strains of the year 2010 approximately 25% on average and approximately 19% in the strains of the year 2000 On average (Figure 3B) (*p* < 0.012). The observed increase was produced especially in genes related to the R–M system and to those related to new phage resistance mechanisms and the CRISPR-Cas system (*p* < 0.0119 and *p* < 0.0144, respectively).

### Clustered Regularly Interspaced Short Palindromic Repeats Arrays

One hundred eighty putative predicted arrays were found (without filtering) using metaCRT in the 18 genomes of ST-2 *A. baumannii* clinical strains (Supplementary Table 3). After the complete process of filtering designed by Shmakov et al. (2020) and removing the low-complexity sequences (Zhang and Ye, 2017), only 40 CRISPR arrays were selected (Table 1): 18 CRISPR arrays were present in the 2000 strains and 22 in the 2010 strains. All the strains, except in the Ab161_GEIH-2000 strain, presented at least one CRISPR array. Surrounding proteins nearby the selected CRISPR arrays are also attached (Supplementary Table 4).

**TABLE 1:** CRISPR arrays present in genomes of 18 *A. baumannii* clinical strains.

Strain	Contig	Size	Start	Stop	Repeat	No. of spacers
Ab33_GEIH-2010	MSMK01000003	160	10,462	10,622	ATTTTGAATTTAAAA	4
Ab33_GEIH-2010	MSMK01000187	198	18,280	18,478	ACAAAAGAAAAAT	4
Ab49_GEIH-2010	MSMM01000317	96	1,114	1,210	TCATTTTGCTGTTGTT	2
Ab49_GEIH-2010	MSMM01000323	198	78	276	ACAAAAGAAAAAT	4
Ab49_GEIH-2010	MSMM01000347	122	367	489	TTTTAAATTCAAAA	3
Ab54_GEIH-2010	MSML01000240	198	3,914	4,112	AATTTTCTTTTCT	4
Ab54_GEIH-2010	MSML01000469	96	1,108	1,204	TCATTTTGCTGTTGTT	2
Ab54_GEIH-2010	MSML01000525	164	8,017	8,181	ATATATTTTTGA	3
Ab76_GEIH-2010	MSLY01000008	96	835	931	TCATTTTGCTGTTGTT	2
Ab76_GEIH-2010	MSLY01000677	198	3,369	3,567	AATTTTCTTTTCT	4
Ab76_GEIH-2010	MSLY01000708	164	714	878	ATATATTTTTGA	3
Ab103_GEIH-2010	MSLX01000655	160	9,148	9,308	ATTTTGAATTTAAAA	4
Ab103_GEIH-2010	MSLX01000266	164	2,680	2,844	ATATATTTTTGA	3
Ab103_GEIH-2010	MSLX01000388	96	1,108	1,204	TCATTTTGCTGTTGTT	2
Ab103_GEIH-2010	MSLX01000506	198	55	253	ACAAAAGAAAAAT	4
Ab104_GEIH-2010	MSMA01000019	96	1,450	1,546	TCATTTTGCTGTTGTT	2
Ab104_GEIH-2010	MSMA01000107	160	4,402	4,562	TTTTAAATTCAAAAT	4
Ab104_GEIH-2010	MSMA01000246	164	10,815	10,979	ATATATTTTTGA	3
Ab105_GEIH-2010	LJHB01000001	198	125,508	125,706	ACAAAAGAAAAAT	4
Ab105_GEIH-2010	LJHB01000010	292	7,321	7,613	TAAAATAATTTTAA	5
Ab121_GEIH-2010	MSLZ01000141	198	4,992	5,190	AATTTTCTTTTCT	4
Ab122_GEIH-2010	MSMD01000782	164	711	875	ATATATTTTTGA	3
Ab155_GEIH-2000	LJHA01000001	198	125,512	125,710	ACAAAAGAAAAAT	4
Ab155_GEIH-2000	LJHA01000002	292	7,323	7,615	TAAAATAATTTTAA	5
Ab158_GEIH-2000	MSMC01000196	198	4,027	4,225	AATTTTCTTTTCT	4
Ab158_GEIH-2000	MSMC01000525	136	868	1,004	ATTTTTTAATATTTA	3
Ab166_GEIH-2000	MSMG01000383	86	859	945	AAATAGCCTAAGC	2
Ab166_GEIH-2000	MSMG01001001	198	293	491	ACAAAAGAAAAAT	4
Ab166_GEIH-2000	MSMG01000974	79	1,310	1,389	TCTGCTGTCGGAAA	2
Ab166_GEIH-2000	MSMG01001128	194	304	498	ACGACGTGGACGATCTTC	3
Ab169_GEIH-2000	MSMF01000039	96	797	893	TCATTTTGCTGTTGTT	2
Ab169_GEIH-2000	MSMF01000336	198	152	350	ACAAAAGAAAAAT	4
Ab175_GEIH-2000	MSMI01000153	79	8,115	8,194	TTTCCGACAGCAGA	2
Ab175_GEIH-2000	MSMI01000682	86	2,355	2,441	AAATAGCCTAAGC	2
Ab177_GEIH-2000	MSME01000459	198	215	413	ACAAAAGAAAAAT	4
Ab183_GEIH-2000	MSMJ01000620	96	1,077	1,173	TCATTTTGCTGTTGTT	2
Ab183_GEIH-2000	MSMJ01000380	198	78	276	ACAAAAGAAAAAT	4
Ab192_GEIH-2000	MSMH01000263	96	1,139	1,235	TCATTTTGCTGTTGTT	2
Ab192_GEIH-2000	MSMH01000273	157	0	157	TTGAATTTAAAA	4
Ab192_GEIH-2000	MSMH01000395	198	21,634	21,832	ACAAAAGAAAAAT	4

**FIGURE 4:**
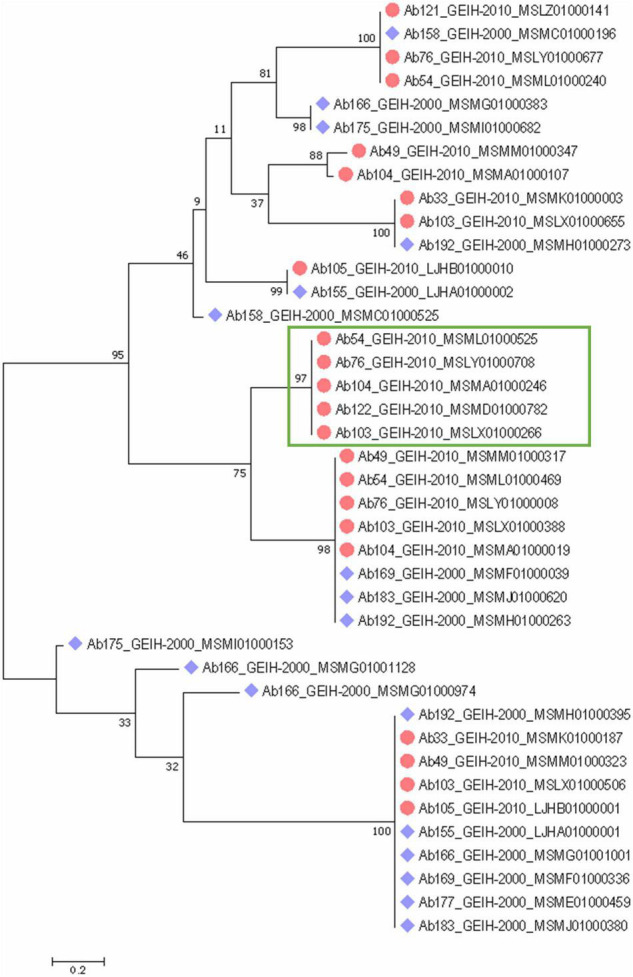
Phylogenetic classification of CRISPR arrays detected in 18 genomes of *A. baumannii* ST-2 clinical strains through a maximum-likelihood tree with suggested model by MEGA analysis Tamura 3-parameter with uniform rates among sites and a bootstrap of 100. Blue rhomboid indicates that strain belongs to 2000 collection. Red circles to 2010 collection. Green rectangle indicates five CRISPR arrays only detected in 2010 strains.

A phylogenetic tree of the complete CRISPR array sequences was constructed (Figure 4) and showed an equal distribution of the spacers between the strains of the 2 years. Some of the spacers were predicted to be the same even in strains of different year collections. Few of the arrays were unique with respect to the other, such as the present in the 2000 strains Ab158_GEIH-2000_MSMC01000525, Ab166_MSMG01000383, Ab166_MSMG01000974, Ab166_MSMG01001128, Ab175_MSMI01000153, or Ab175_ MSMI01000682. However, there were five CRISPR arrays grouped that only were represented in the 2010 strains (Figure 4).

### Cas-Related Proteins

When HMM against Cas-known, Cas-related, and CRISPR-associated protein families was used, 705 Cas-related proteins were identified in the 18 genomes: 341 Cas-related proteins were detected in the 2000 strains and 364 in the 2010 strains (Supplementary Table 5). Most of them were identified as DEAD/DEAH box helicase (207 of the total) and as type III Restriction Unit Res III (195 of 705). The vast majority of them were located next to proteins whose predicted function does not match with a Cas protein function or to proteins whose function was unknown. Other Cas-related proteins were close in the same contig, thereby giving us a clue to help identify a functional Cas cluster. For example, in the contig MSLX01000260 from the Ab103_GEIH-2010 strain, a putative Helicase_C protein (OLV37994.1) and a Cas_St_Csn2 protein (OLV37998.1) were only of 2 ORF distance between them. However, the function of the surrounding proteins was hypothetical, thus hindering the identification process as a Cas cluster.

## Discussion

In clinical laboratories, genomics is rapidly being developed and utilized to track antibiotic resistance. As a result, it is critical to explore how to detect and avoid phage-resistant strains if a treatment based on phages was going to be applied, using WGS metadata analysis. In this study, we looked for genes linked to phage resistance in 18 clinical strains of *A. baumannii.* We constructed a database with genes based in the public PADS database, as it is a complete database about prokaryotic antiviral defense systems so far, as well as being collecting newly discovered types of defense systems to the BIG Data Center (BIG Data Center Members, 2019; Zhang et al., 2020). As mutations in bacterial membrane proteins or LPS is a primary defense due to causing a lower fitness cost to the bacteria (van Houte et al., 2016), and they should potentially also be included to track phage resistance in WGS because they proved appearance in the application of cocktail of phages (Hesse et al., 2020; Yang et al., 2020), the aim was to identify defense genes whose function were putatively only related to phage resistance mechanisms. In this case, the high number of genes made us establish groups to simplify the results of the blast hits. We also tried to identify the presence of CRISPR-Cas systems by separating the search in CRISPR arrays and Cas proteins.

A difference between the presence of phage-resistant genes in the 2010 strains and the 2000 strains was observed, with a higher presence of genes related to the R–M system and CRISPR-Cas system and lower TA-related genes. The natural reciprocal selection pressure between host bacteria and phage increases the infectivity of the phage and the phage resistance in the bacterium side (Hampton et al., 2020). In fact, phage populations are ubiquitous at body surfaces such as lungs, intestines, or skin, and they outnumber bacteria at least 10-fold (Batinovic et al., 2019). The study of the mobilome (plasmids and bacteriophages) in these same 18 genomes belonging to the *A. baumannii* clonal group ST-2 in 2018 (López et al., 2018) connects the presence of functional quorum-sensing/quorum-quenching network in those cells with a higher presence of complete prophages in the 2010 collection. As it is pointed out, the representative strain Ab105_GEIH-2010 (representative of the Ab_GEIH-2010 group of strains) owns two complete prophages: Ab105-1Φ and Ab105-2Φ. One of them, Ab105-2Φ, was turned into a mutant lytic phage, and its phage infectivity was tested as host range and efficiency of plating against some *A. baumannii* clinical strains, in which some of the strains of this study are included (Blasco et al., 2019). By contrast, none of the Ab_GEIH-2000 group possess any complete prophage. For this reason, the acquisition of phage-resistant genes is correlated with a higher presence of complete prophages in the strains of 2010 in comparison with those of 2000.

Defense systems are regularly obtained by bacteria and archaea through horizontal gene transfer (HGT) owing to an environmental adaptation of the bacterial communities (van Houte et al., 2016; Koonin et al., 2017). We found a major average of genes acquired by HGT in the 2010 strains (25% were in GIs regions) rather than in the 2000 ones (19% were in GIs regions), especially those genes related to R–M system and CRISPR-Cas. It was demonstrated that only ∼4% of R–M systems are in the core genomes of prokaryotic species, suggesting they are commonly transferred (Oliveira et al., 2014). CRISPR-Cas systems display weak consistency within the core genome, demonstrating the prevalence of the HGT spreading this system (Oliveira et al., 2014; Makarova et al., 2015). The R–M system and the CRISPR-Cas system commonly coexist with an elevated contribution to bacterial immunity, and they rarely operate on their own (Dupuis et al., 2013; Oliveira et al., 2014). However, they are far from being perfect in the bacterial resistance, and phage can escape these systems in many different ways, for example, the anti-CRISPR proteins (Labrie et al., 2010; Bondy-Denomy et al., 2013). We also observed a decreasing number of TA-related genes through the years; even their presence in GIs is higher in the 2000 strains than in the 2010s. This could be because the counteradaptation of the phage may be reached by developing antitoxin in the phage genome that inhibits the cell death and thus promote the infection of the phage (Otsuka and Yonesaki, 2012; Wei et al., 2016) or because they could have evolved into Cas proteins of the CRISPR-Cas system, as the TA proteins are considered as ancestors of Cas2 proteins (Makarova et al., 2020). In *A. baumannii*, little is known about defense mechanisms against phage. Another recent genomic bioinformatics analysis, in this case in the sequence type ST374, revealed the presence of a defense island with genes involved in Abi, R–M, BREX, or TA systems (Fedrigo et al., 2021).

Furthermore, we found the CRISPR-Cas genes blast hit results incomplete due to the separation in contig assembly of the genomes, which prevented us from identifying proteins or arrays related to the CRISPR-Cas proteins identified in small contigs (data not shown), and also due to the high diversity of the Cas proteins and the little knowledge about these proteins in clinical strains of *A. baumannii*, which increases the difficulty of identifying these type of proteins (Mangas et al., 2019; Pinilla-Redondo et al., 2020). As a consequence, we examined the presence of CRISPR arrays and Cas proteins separately. We establish a methodology to discard false-CRISPR elements based on the method of Shmakov et al. (2020) and posteriorly completed with a full evaluation of the quality of the CRISPR arrays filtered based on the search of tandem repeats, simple repeats, and their presence on phage genomes (Zhang and Ye, 2017). Secondly, another reason for developing an alternative method is the nature of the multiresistant pathogens, their constant adaptation to different environments, and thus the continuous acquisition of different mobile elements, which provokes the appearance of new CRISPR-Cas yet to be identified (Kamruzzaman and Iredell, 2019). This also fosters and extends the variability in the Cas proteins, complicating their characterization.

Forty CRISPR arrays were found in the 18 *A. baumannii* clinical strains from the ST-2 clone. All of the strains presented at least one CRISPR array except one, Ab161_GEIH-2000. The vast majority of the arrays are shared between the clone ST-2 in both collections, with some exceptions such as the five arrays only found in the 2010 strains. It has been shown that the distribution of the CRISPR-Cas system is MLST-dependent and non-random and thought to be a better discriminating tool than classical MLST in discriminating different *Klebsiella pneumoniae* (Shen et al., 2017; Liao et al., 2020). On the other hand, the detection of different unique CRISPR arrays only in the 2000 strains demonstrates the dynamic interaction of these arrays throughout the years.

All of the CRISPR arrays in this study were without any Cas or putative Cas protein near to them. It was described that these “orphan” arrays belong to unknown CRISPR-Cas systems due to an extremely evolutionarily remote type of CRISPR-Cas (Shmakov et al., 2020). This existence of isolated CRISPR arrays could be explained for four reasons. First, the contig format of the studied genomes could provoke that some arrays are detected in small or incomplete contigs. Secondly, some Cas endonucleases such as Cas1 and/or Cas6 can recognize remote CRISPR arrays (Reimann et al., 2020; Hoikkala et al., 2021). Third, the possibility of some of the unique isolated arrays forming part of an undescribed CRISPR-Cas cluster extremely distant from the ones already characterized may occur (Shmakov et al., 2020). Fourth, the strains may have lost the *cas* genes, thus leaving the isolated arrays (Shmakov et al., 2020). The Cas distribution observed in this work would correspond and complete any of the hypotheses about the explanation of “orphan” CRISPR arrays mentioned before, as the putative Cas proteins hit through the HMM search could form part of a complete Cas cluster. However, as it was said at the end of *Results*, it was impossible to determine *in silico* if the putative Cas detected form part of complete and functional Cas loci.

The localization and characterization of defense systems against phages are necessary steps when designing an effective phage therapy. The WGS combined with an effective bioinformatics strategy would allow us to know what mechanisms the clinical strains have and potentially discard those phages to which bacteria may possess resistance to the treated strain. This study shows the wide presence of genes associated with resistance against phages and their acquisition by GIs for 10 years in clinical *A. baumannii* strains from the same clonal complex ST-2 and the CRISPR arrays present on them. Additional investigations are needed to characterize and discover the molecular mechanisms of phage-resistant genes discovered in this study in *A. baumannii*.

## Data Availability Statement

The original contributions presented in the study are included in the article/Supplementary Material, further inquiries can be directed to the corresponding author/s.

## Author Contributions

AA, LB, ML, OP, IB, LF-G, MG, and CO-C analyzed the results and wrote the manuscript. AM revised the results and the manuscript. MT obtained the research funding and supervised the writing of the manuscript. All authors contributed to the article and approved the submitted version.

## Conflict of Interest

The authors declare that the research was conducted in the absence of any commercial or financial relationships that could be construed as a potential conflict of interest.

## Publisher’s Note

All claims expressed in this article are solely those of the authors and do not necessarily represent those of their affiliated organizations, or those of the publisher, the editors and the reviewers. Any product that may be evaluated in this article, or claim that may be made by its manufacturer, is not guaranteed or endorsed by the publisher.

## Footnotes

1
https://bigd.big.ac.cn/padsarsenal/

2
www.python.org

## Supplementary Material

Supplementary Table 3 Putative predicted arrays (without filtering) using metaCRT in the 18 genomes of ST-2 *A. baumannii* clinical strains (.xls file).

Supplementary Table 4 Adjacent predicted proteins of CRISPR arrays detected.

Supplementary Table 5 Cas-related proteins identified with HMM against a prebuilt database with known Cas proteins and proteins with functions related with Cas proteins (.xls file).
