# Supplementary material for: Genomic Analysis of Molecular Bacterial Mechanisms of Resistance to Phage Infection
Source: Front Microbiol. 2022 Feb 17;12:784949. doi: 10.3389/fmicb.2021.784949 (PMC8891609; doi:10.3389/fmicb.2021.784949)
Supplement: Supplementary file 4 [file Table_1.docx]

|  | Ab33_  GEIH-2010 | Ab49_  GEIH-2010 | Ab54_  GEIH-2010 | Ab76_  GEIH-2010 | Ab103_GEIH-2010 | Ab104_GEIH-2010 | Ab105_GEIH-2010 | Ab121_GEIH-2010 | Ab122_GEIH-2010 | Ab155_GEIH-2000 | Ab158_GEIH-2000 | 161_  GEIH-2000 | Ab166_GEIH-2000 | Ab169_GEIH-2000 | Ab175_GEIH-2000 | Ab177_GEIH-2000 | Ab183_GEIH-2000 | Ab192_GEIH-2000 |
| --- | --- | --- | --- | --- | --- | --- | --- | --- | --- | --- | --- | --- | --- | --- | --- | --- | --- | --- |
| ABI | 11 | 9 | 11 | 12 | 11 | 11 | 12 | 12 | 10 | 11 | 8 | 11 | 5 | 11 | 11 | 9 | 11 | 11 |
| TA | 35 | 35 | 32 | 31 | 32 | 32 | 35 | 33 | 33 | 32 | 32 | 30 | 26 | 36 | 36 | 29 | 36 | 36 |
| RM | 50 | 54 | 46 | 48 | 53 | 53 | 40 | 53 | 46 | 36 | 44 | 48 | 40 | 42 | 42 | 28 | 42 | 42 |
| CRISPR-CAS | 1 | 0 | 1 | 0 | 2 | 2 | 0 | 1 | 1 | 0 | 0 | 1 | 0 | 0 | 0 | 0 | 0 | 0 |
| NEW | 75 | 69 | 68 | 59 | 64 | 63 | 59 | 61 | 62 | 57 | 60 | 58 | 54 | 63 | 67 | 52 | 66 | 63 |
| Total genes | 3988 | 3861 | 3946 | 3908 | 3922 | 3947 | 3923 | 3936 | 3875 | 3759 | 3898 | 3864 | 3674 | 3745 | 3785 | 2811 | 3899 | 3688 |

**SUPPLEMENTARY MATERIAL**

**Table 1.** Absolute number of each phage resistance genes group in 18 genomes of *A. baumannii* clinical strains.
