# Supplementary material for: Genomic Analysis of Molecular Bacterial Mechanisms of Resistance to Phage Infection
Source: Front Microbiol. 2022 Feb 17;12:784949. doi: 10.3389/fmicb.2021.784949 (PMC8891609; doi:10.3389/fmicb.2021.784949)
Supplement: Supplementary file 5 [file Table_2.docx]

|  | Ab33_  GEIH-2010 | Ab49_  GEIH-2010 | Ab54_  GEIH-2010 | Ab76_  GEIH-2010 | Ab103_GEIH-2010 | Ab104_GEIH-2010 | Ab105_GEIH-2010 | Ab121_GEIH-2010 | Ab122_GEIH-2010 | Ab155_GEIH-2000 | Ab158_GEIH-2000 | Ab161_GEIH-2000 | Ab166_GEIH-2000 | Ab169_GEIH-2000 | Ab175_GEIH-2000 | Ab177_GEIH-2000 | Ab183_GEIH-2000 | Ab192_GEIH-2000 |
| --- | --- | --- | --- | --- | --- | --- | --- | --- | --- | --- | --- | --- | --- | --- | --- | --- | --- | --- |
| ABI | 1 | 1 | 1 | 1 | 1 | 2 | 3 | 2 | 1 | 2 | 1 | 0 | 0 | 1 | 1 | 2 | 2 | 1 |
| TA | 5 | 6 | 4 | 2 | 3 | 5 | 7 | 4 | 3 | 4 | 5 | 5 | 0 | 5 | 10 | 0 | 4 | 2 |
| RM | 26 | 26 | 25 | 18 | 27 | 23 | 12 | 26 | 23 | 12 | 19 | 21 | 16 | 18 | 20 | 9 | 11 | 22 |
| CRISPR-CAS | 1 | 0 | 1 | 0 | 2 | 2 | 0 | 1 | 1 | 0 | 0 | 1 | 0 | 0 | 0 | 0 | 0 | 0 |
| NEW | 10 | 9 | 9 | 8 | 6 | 9 | 10 | 10 | 11 | 8 | 8 | 5 | 3 | 6 | 5 | 5 | 0 | 7 |
| Total genes in GIs | 43 | 42 | 40 | 29 | 39 | 41 | 32 | 43 | 39 | 26 | 33 | 32 | 19 | 30 | 36 | 17 | 25 | 33 |

**SUPPLEMENTARY MATERIAL**

**Table 2.** Absolute number of genes predicted in the Genomic Island (GIs).
